# Incidence and Clinical Outcomes of COVID‐19 in Maintenance Hemodialysis Patients in Libya: A Prospective Descriptive Study

**DOI:** 10.1155/crdi/4419576

**Published:** 2026-01-19

**Authors:** Nada Elgriw, Eman Gusbi, Halla Elshwekh, Jamal Elcosbi, Inas Alhudiri, Ezedeen M. Belhaj, Aymen M. Alamin, Adam Elzagheid, Nabil Enattah

**Affiliations:** ^1^ Microbiology Department, Biotechnology Research Center, Tripoli, Libya; ^2^ Physiology Department, University of Tripoli, Tripoli, Libya, uot.edu.ly; ^3^ Genetic Engineering Department, Biotechnology Research Center, Tripoli, Libya; ^4^ Tripoli Hemodialysis Center, Tripoli, Libya

**Keywords:** chest, COVID-19, hemodialysis, PCR

## Abstract

Hemodialysis patients are at a greater risk of severe disease from COVID‐19. Of the 600 maintenance hemodialysis patients who were regular attendees at the Tripoli Hemodialysis Center in Tripoli, Libya, 12 patients tested positive for SARS‐CoV‐2 infection. The patients’ ages ranged from 48 to 80 years. Three were female (25%), and four (33.3%) reported prior contact with a confirmed case. The most common symptoms were fever (66.7%), dry cough (66.7%), dyspnea (91.7%), and fatigue (83.3%). Chest computed tomography revealed radiological features consistent with COVID‐19 pneumonia, including ground‐glass opacities and pulmonary consolidation in all patients. Four of the patients died (33.3%). COVID‐19 represents a significant comorbidity in maintenance hemodialysis patients and is associated with a notably high mortality rate. In the absence of specific operational guidelines, tailored protocols should be developed, or existing guidelines—such as those from the CDC—should be adapted to fit the local healthcare context.

## 1. Introduction

In December 2019, the world experienced the first outbreak of the highly contagious COVID‐19 disease, which is a serious threat to the health and life of elderly people and those with certain chronic diseases, including patients with kidney failure. In references [1–5] many maintenance hemodialysis (MHD) patients are of an older age, and many of them have comorbidities, such as hypertension, diabetes, cardiovascular disease, lung disease, and immune‐compromised status. MHD patients may be at increased risk for COVID‐19 infection and its complications. They have worse outcomes [4–10]. Higher mortality has been observed among hospitalized COVID‐19 patients with chronic kidney disease (CKD), especially those in stages 3–5 CKD, than patients without CKD. Stages 3–5 CKD patients have an in‐hospital mortality rate similar to that of hemodialysis patients, which may be partly due to age similarity and comorbidity burden [5, 11–14].

No study on COVID‐19 infection in patients on hemodialysis or on how hemodialysis patients are being handled seems to have been done in Libya. A search of PubMed and Google Scholar with the search string ‘COVID + dialysis + Libya’ yielded no hits. It is important to pay attention to these particularly vulnerable patients. This prospective descriptive study reports on 12 MHD patients attending the main hemodialysis center in Tripoli and identified by reverse‐transcriptase polymerase chain reaction (RT‐PCR) screening to be positive for SARS‐CoV‐2.

## 2. Patients and Methods

The study was a descriptive, prospective, cohort type conducted at the Tripoli Hemodialysis Center (Tripoli, Libya) which is considered the largest hemodialysis center in Libya. This center loads 600 MHD. The study included all MHD attending this Center over a period of three months from September to November 2020. PCR screening for COVID‐19 of all MHD patients began after the first infected patient was identified at the Center in September 2020. Of the 600 MHD patients, 12 patients developed COVID‐19 pneumonia, confirmed by the RT‐PCR test for SARS‐CoV‐2 on nasopharyngeal swabs.

Data collected from medical file records which included demographic characteristics, such as age, sex, contact with infected persons, family members affected, dialysis vintage by years, if the patient is diabetic or not, smoking, the cause of end stage renal disease, blood group and the symptoms that appeared such as fever, dry cough, dyspnea, fatigue, diarrhea, abdominal pain, also laboratory findings of 12 MHD patients which infected with SARS‐CoV2 were collected which include leukocytes count, neutrophils count, lymphocytes count, erythrocytes, hemoglobin, platelets, heart rate, respiratory rate, urea, creatine, oxygen saturation, C‐reactive protein, D‐Dimer, ferritin, albumin, triglycerides, total bilirubin, gamma‐glutamyl transferase, alanine aminotransferase, blood urea nitrogen, chloride (Cl‐), potassium (K+), sodium (Na+), creatine kinase, low‐density lipoprotein, alkaline phosphatase, glucose, direct bilirubin, lactate dehydrogenase, high‐density lipoprotein, total bilirubin.

Computerized tomography of the chest was performed only on those shown to be positive by RT‐PCR.

### 2.1. Ethical Considerations

Ethical approval was obtained from Bioethics Committee at Biotechnology Research Center (BEC‐BTRC 18‐2021).

### 2.2. Statistical Analysis

Statistical analysis of data were performed using SPSS Software Version 26 (International Business Machines Corporation [IBM], New York, USA) for windows. Data are expressed as number, range, and mean ± standard deviation or percent frequency, as appropriate. The chi‐square test was used to analyze correlation significance (< 0.05).

## 3. Results

The profiles of 12 COVID‐19 positive MHD patients were evaluated; the results showed that three of them were females, and their ages ranged from 48 to 80 years, with the mean age of 64 years. Four of them had a history of contact with an infected person, and four had family members infected with COVID‐19. Eight were diabetic, and one was a nonsmoker. Four of the patients had been dialyzed for 4 years and one for 5 years. The cause of end‐stage renal disease was hypertensive nephropathy in eight patients, diabetic nephropathy in three, and chronic nephritis in one.

All of whom were symptomatic; the most common symptoms reported at the onset of illness were dyspnea (11/12), fatigue (10/12), fever (8/12) and dry cough (8/12). The less common symptoms were diarrhea (4/12) and abdominal pain (6/12). No patient complained of rhinorrhea, sore throat, or myalgia (Table 1).

**Table 1:** Clinical characteristics of the 12 maintenance hemodialysis patients infected with SARS‐CoV‐2.

Patient no.	1	2	3	4	5	6	7	8	9	10	11	12
Age (years)	70	56	64	73	80	67	62	48	64	58	62	66
Sex	M	M	M	F	M	F	M	F	M	M	M	M
Contact with infected person	No	Yes	No	No	Yes	No	Yes	No	No	Yes	No	No
Family members affected	No	Yes	No	No	Yes	No	Yes	No	No	Yes	No	No
Dialysis vintage (years)	4	2	5	7	2	4	7	3	4	6	4	6
Diabetes	Yes	Yes	No	Yes	Yes	Yes	No	Yes	Yes	No	No	Yes
Fever	Yes	Yes	Yes	Yes	No	No	Yes	No	Yes	No	Yes	Yes
Dry cough	Yes	No	Yes	Yes	No	Yes	Yes	No	Yes	Yes	No	Yes
Dyspnea	No	Yes	Yes	Yes	Yes	Yes	Yes	Yes	Yes	Yes	Yes	Yes
Fatigue	Yes	No	Yes	Yes	Yes	Yes	Yes	Yes	Yes	Yes	No	Yes
Diarrhea	No	No	Yes	No	Yes	No	No	No	Yes	No	Yes	No
Abdominal pain	No	No	Yes	Yes	Yes	Yes	No	No	Yes	No	Yes	No
Leukocytes (10^3^/μL)	7.4	6.7	5.4	9.7	21.9	7.5	12.4	7.4	5.4	6.7	17.3	7.4
Neutrophils (%)	6.1	19.8	2.8	7.5	16.8	5.9	10.7	6.1	2.8	19.8	18.2	6.1
Lymphocyte (%)	0.6	0.3	2.3	1.7	0.4	0.9	0.7	0.6	2.3	0.3	0.3	0.6

From the laboratory finding of the 12 COVID‐19‐positive MHD patients, four patients had blood group A− and three were O+. Two patients were A+, two AB+, and one O−.

White blood cell counts were elevated in 3 of 12 patients (mean 9.6103/μL ± 5.1 SD). Neutrophil counts were elevated in 10 of 12 patients (range 40%–80%). Lymphocytopenia was observed in 8 of 12 patients, as shown in Table 1.

The mean hemoglobin concentration of the patients was 11.1 g/dL ± 1.9 SD. The mean red blood cell count was 4.0106/μL ± 0.7 SD. The mean platelet count was 211.6103/μL ± 113.4 SD. The D‐Dimer values of half of the patients were within normal range and the others were above normal range, with an overall mean value of 499.2 μg/mL ± 250.4 SD. The mean values and ranges of the entire laboratory are shown in Table 2.

**Table 2:** Laboratory findings of 12 maintenance hemodialysis patients with COVID‐19.

	*n*	Range	Mean	SD
Age (years)	12	48.0–80.0	64.2	8.3
Leukocyte count(10^3^/μL)	12	5.4–21.9	9.6	5.1
Neutrophils (10^3^/μL)	12	2.8–19.8	10.2	6.6
Lymphocytes (10^3^/μL)	12	1.7–9.0	4.4	2.3
Erythrocytes (10^6^/μL)	12	2.1–4.9	4.0	0.7
Hemoglobin (g/dL)	12	7.0–14.0	11.1	1.9
Platelets (10^3^/μL)	12	106.0–498.0	211.6	113.4
Heart rate (beats/min)	12	67.0–150.0	96.8	22.3
Respiratory rate (breaths/min)	12	12.0–79.0	28.4	18.3
Urea (mg/dL)	12	8.5–147.0	72.3	39.8
Creatinine (mg/dL)	12	1.7–23.0	10.1	7.6
pH	12	7.0–7.5	7.4	0.2
PCO_2_ (mmHg)	12	31.2–93.7	44.7	18.4
PO_2_ (mmHg)	12	37.5–192.0	104.8	49.1
Oxygen saturation (%)	12	59.7–99.0	91.6	11.3
C reactive protein (mg/L)	12	99.4–480.5	211.6	96.1
D‐Dimer (μg/mL)	12	147.0–951.0	499.2	250.4
Ferritin (ng/mL)	12	731.0–4320.0	1915.2	1056.3
Albumin (g/dL)	12	2.3–13.2	5.2	3.5
Triglycerides (mg/dL)	12	106.0–208.0	156.7	31.6
Total bilirubin (mg/dL)	12	0.3–1.7	0.7	0.4
Gamma‐glutamyl transferase (U/L)	12	32.0–86.0	55.0	14.8
Alanine aminotransferase (units/L)	12	11.9–288.1	57.5	78.9
Blood urea nitrogen (mg/dL)	12	10.9–164.0	51.4	48.1
Cl^─^ (mEQ/L)	12	33.0–127.0	96.6	24.7
K^+^ (mmol/L)	12	1.7–6.7	4.3	1.3
Na^+^ (mmol/L)	12	62.2–163.9	134.0	26.3
Creatine kinase (units/L)	12	27.0–429.0	141.9	114.3
Low‐density lipoprotein (mg/dL)	12	50.0–557.0	113.1	140.4
Alkaline phosphatase (U/L)	12	32.0–224.0	84.8	50.5
Glucose (mg/dL)	12	126.0–804.0	319.8	220.9
Direct bilirubin (mg/dL)	12	0.1–1.2	0.6	0.4
Lactate dehydrogenase (μ/L)	12	231.0–547.0	390.1	111.5
High‐density lipoprotein (mg/dL)	12	7.0–45.0	26.9	14.2
Total bilirubin (mg/dL)	12	0.0–1.3	5.9	2.1

Abbreviation: SD, standard deviation.

**Figure 1:**
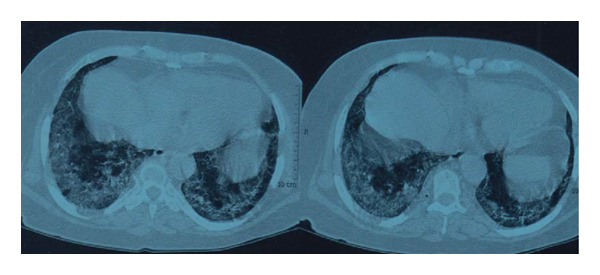
Chest high‐resolution computed tomography of a maintenance hemodialysis patient with SARS‐CoV‐2 infection.

**Figure 2:**
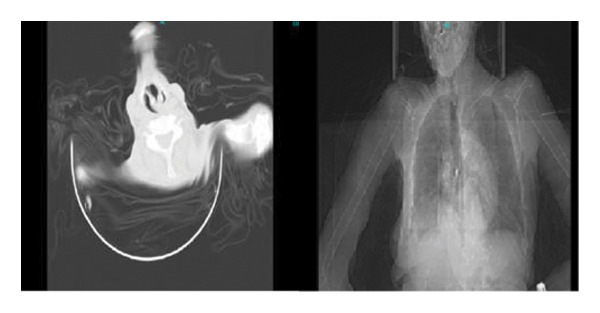
Chest high‐resolution computed tomography of a patient PCR‐positive for SARS‐CoV‐2 with cycle threshold 12 showing radiological features of atypical viral pneumonia CORADS 5 (reporting and data system CATEGORY 5) [4].

Abnormalities seen in high‐resolution computed tomography images of the chest were detected in all 12 patients (examples are shown in Figures 1 and 2). The radiological features are consistent with COVID‐19 pneumonia CORADS‐5, which represents the category with the highest suspicion of COVID‐19. Multiple, scattered, peripheral bi‐basal areas of ground‐glass opacities and consolidation are present, with areas of traction bronchiectasis and dilated subpleural pulmonary vessels.

Among the 12 patients, four died. These patients had significantly higher ferritin levels (ranging 1500–4000 μg/L) and elevated D‐Dimer values compared to survivors. Three of the deceased were diabetic. While statistical tests are limited by sample size, the trends suggest possible associations between hyperinflammation markers and mortality.

Inferential statistical analysis revealed that ferritin levels were significantly higher among deceased patients (Mann–Whitney U test, *p* = 0.048).

D‐Dimer levels showed a nonsignificant trend (*p* = 0.109), and diabetes was not significantly associated with mortality (chi‐square test, *p* = 1.0).

Although blood group A was more frequent among patients with severe lung involvement (as shown in CT scans), statistical correlation could not be established due to the limited sample size. This observation warrants further investigation in larger cohorts.

## 4. Discussion

In this prospective descriptive study, we evaluated the incidence and clinical outcomes of COVID‐19 among 600 MHD patients at the Tripoli Hemodialysis Center. A total of 12 patients (2.0%) tested positive for SARS‐CoV‐2. The most common clinical manifestations were dyspnea, fatigue, fever, and dry cough. Radiological findings revealed ground‐glass opacities and lung consolidation in all cases, which are consistent with COVID‐19 pneumonia.

Our study observed a mortality rate of 33.3%, comparable to reports from other hemodialysis cohorts worldwide, where mortality has ranged between 30% and 35% [14, 15]. This underscores the high vulnerability of MHD patients to severe COVID‐19 outcomes.

Importantly, deceased patients demonstrated significantly elevated ferritin levels compared to survivors (*p* = 0.048), suggesting that ferritin may serve as a potential biomarker of disease severity in this population. Elevated D‐Dimer levels were also observed among deceased patients, although statistical significance was not reached (*p* = 0.109), likely due to the small sample size. Similarly, while diabetes was more frequent among deceased patients, no significant association with mortality was detected (*p* = 1.0). These findings highlight the need for larger studies to confirm the role of inflammatory markers and comorbidities in predicting outcomes among dialysis patients.

Regarding blood type, we noted a higher frequency of blood group A among patients with severe lung involvement. However, due to the limited number of cases and lack of statistical significance, this observation should be interpreted with caution and requires further investigation in larger cohorts.

This study has several limitations. The most important are the small sample size (only 12 patients) and the absence of a control group of nondialysis COVID‐19 patients, which restrict the generalizability and depth of comparative analysis. Nevertheless, our findings contribute valuable preliminary data on COVID‐19 among dialysis patients in Libya, a population for which no previous reports were available.

Given the fragile health status of MHD patients and their need for frequent in‐center treatment, dialysis facilities must implement strict infection‐prevention protocols and adapt international guidelines to the local healthcare context in order to mitigate risks.

The patients who had CKD had a higher mortality rate than heart disease patients [16]. In one small study, the mortality rate among hemodialysis patients was 30.0% [17], which resembles our observation of 33.3% mortality among MHD patients. Moreover, our observation that 75% of the patients were males is in agreement with the findings of the Ozturk study [16].

It has been confirmed that T‐cell immunity is a key factor in recovery from SARS‐CoV‐2 infection [18]. Because uremia status is associated with extensive impairment of lymphocyte and granulocyte function, an abnormal immune system may alter the response to SARS‐CoV‐2 infection. References [19, 20] show particular concern given the densely populated and busy nature of dialysis facilities, which create a high risk of transmission of the virus. However, in our dialysis center, the virus does not seem to have spread widely.

Of the 12 patients, 4 (33.3%) died. These individuals presented with markedly elevated inflammatory markers, including D‐Dimer (above 500 μg/L) and ferritin (1500–4000 μg/L). These biomarkers have previously been linked to poor prognosis in COVID‐19. Of who had diabetes and in general were experiencing fatigue, dizziness, fever, and polyuria. Whereas the normal range for ferritin is 15–350 μg/L. D‐Dimer is a biomarker for COVID‐19 severity and a death risk factor. While the normal range is < 0.5 mg/L, the patient who died had D‐Dimer levels of up to 400 mg/L. Three of the deceased patients had diabetes, a known risk factor for severe outcomes. While no formal statistical comparison was conducted due to small sample size, this pattern aligns with previous literature.

## 5. Conclusions

Among 600 maintenance hemodialysis patients at the Tripoli Hemodialysis Center, 12 (2.0%) tested positive for COVID‐19. The most common symptoms were dyspnea, fatigue, fever, and dry cough, with radiological findings of ground‐glass opacities and lung consolidation in all patients.

Lymphocytopenia was frequently observed. The mortality rate was high (33.3%), and ferritin levels were significantly elevated among deceased patients, suggesting its potential role as a severity biomarker.

These findings highlight the substantial risk that COVID‐19 poses to hemodialysis patients and emphasize the urgent need for tailored preventive and management strategies in this vulnerable group.

Among the 12 infected patients, the most common symptoms were dyspnea, fatigue, fever, and dry cough. Radiological findings revealed ground‐glass opacities and lung consolidation in all patients.

Lymphocytopenia was frequent, and 33.3% of patients died.

Statistical analysis revealed significantly elevated ferritin levels among deceased patients (*p* = 0.048), suggesting it may serve as a potential biomarker of severity.

Due to their clinical fragility and need for frequent in‐center care, MHD patients require stringent protective measures.

This study’s small sample size and single‐center scope limit generalizability, but it highlights critical risk factors in a vulnerable population and underscores the need for tailored clinical guidelines.

## Disclosure

A preprint has previously been published.^23^

## Conflicts of Interest

The authors declare no conflicts of interest.

## Author Contributions

Eman Gusbi, Nada Elgriw, Halla Elshwekh conceived the study and wrote the manuscript.

Ezedeen M. Belhaj, Aymen M. Alamin: 2 physicians in Tripoli Hemodialysis Center which help us to deal with MHD patients participated in data collection.

Adam Elzagheid, Nabil Enattah: 2 physicians the head of Biotechnology Research Center in which PCR undergone.

Jamal Elcosbi, Inas Alhudiri: Researcher in Biotechnology Research Center which give us the materials for testing.

## Funding

No financial support was received for this study.
